# "You're an inmate just deal with it": experiences of formerly incarcerated individuals in New York City during the COVID-19 pandemic

**DOI:** 10.1186/s40352-025-00391-z

**Published:** 2025-12-17

**Authors:** Erinn Bacchus, Sergio Rivera Rodriguez, Keith Gordon, Naomi Zewde

**Affiliations:** 1 College of Health, Lehigh University, Bethlehem, United States; 2 CUNY Graduate School of Public Health and Health Policy, New York, United States; 3 UCLA Fielding School of Public Health, Los Angeles, United States

**Keywords:** Incarceration, COVID-19, Power dependent, Jails, Healthcare, Qualitative, Public health, Health policy, Service delivery

## Abstract

The onset of the COVID-19 pandemic posed uniquely dire challenges in New York City’s jails. Rates of infection and fatality well exceeded those of the general population as the virus spread through congregate residences, heightening the city's status as an early epicenter. Using an inductive, grounded theory approach, we conduct 12 in-depth interviews with persons incarcerated in these jails early in the pandemic to elicit their perspectives on the carceral system’s ability to deliver healthcare services and a safe residence. Participants describe concerns of overcrowding, insufficient protective equipment, and difficulty accessing healthcare through a lens of interpersonal hierarchy, illustrating how their relationships with one another and with jail staff determined their access to public health measures. The data highlight how incarceration revokes individuals’ autonomy and imposes a power dependent, hierarchical relational structure. That context complicates or precludes the development of mutual trust needed for effective public health interventions. We posit entrusting external entities with health communication during a disease outbreak as a mitigation strategy.

## Introduction

The onset of the COVID-19 pandemic brought dangerous and chaotic conditions, perhaps nowhere more so than for persons incarcerated in New York City’s correctional facilities. The city was an early epicenter of the pandemic at a time when supply shortages turned Central Park into a make-shift morgue and had hospital personnel don trash-bags as personal protective equipment (PPE) (Liveris et al., 2022; Rosner & Sheehy, 2020). Jails were a perfect storm of contagion: congregate residences shared across dozens of strangers and with a revolving door of incarcerated persons, visitors, and staff churning in and out daily (Barnert et al., 2020). Relative to prisons, jail conditions only presented heightened opportunity for contagion where, in contrast to prisons, persons are admitted for shorter sentences, no more than a year, or are awaiting trial (Dholakia, 2023). Exacerbating its consequences, incarcerated persons are drawn from socially and economically disinvested communities, raising the risk of serious prognosis or death for these individuals and those same communities to which they return (Western & Pettit, 2010).

The problem is not only grave; it is large. The US incarcerates more people than any country in the world, with nearly two million persons behind bars (Gramlich, 2021; Sawyer & Wagner, 2023). Epidemiologic studies count at least 650,000 infections and almost 3,000 deaths from COVID among incarcerated persons (Brinkley-Rubinstein & Nowotny, 2022). The rate of transmission was 5.5 times, and the rate of death 3 times, that of the general population in the early months of the pandemic (Saloner et al., 2020). By the end of June 2020, jails and prisons accounted for 16 of the country’s top 20 COVID-19 clusters (Nelson & Kaminsky, 2020). Moreover, even these indicators of pervasive spread represent an underestimate as many jails and prisons opted not to collect or share COVID-related data with officials or the public.

Journalists have reported alarming stories that offer a glimpse behind the numerical accounting. For example, a sixty-four-year-old man died mere “minutes” before an emergency compassionate release could be carried out; multiple others contracted, and died from, COVID while in custody for failing parole violations like being late to meet their parole officer amidst the chaos of the pandemic (Keogh & McShane, 2021; Ransom, 2020). What remains unclear from these disturbing accounts, however, is the extent to which the hierarchical nature of relationships inside carceral settings contributed to the devolution of one’s health and daily experiences during the COVID-19 pandemic.

In this study, we elicit and examine the experiences of persons incarcerated on Riker’s Island, New York City’s largest jail complex, at a time of substantial uncertainty, March 2020. This study contributes to an emerging body of research explicating the effects of the COVID-19 pandemic on the experiences of incarcerated persons. This literature documents experiences such as the difficulties of individuals returning home, managing substance use disorders upon release, perceived risk while incarcerated in high-security prisons, and mitigation strategies implemented in carceral settings.(Abrams et al., 2023; Bono et al., 2023; Pettus-Davis et al., 2021; Pyrooz et al., 2020; Vuolo et al., 2023) Additionally, we build on work interviewing administrators and staff of jails, who relayed concerns of underfunding in carceral health systems; and on the journalism that sought to expose particularly egregious outcomes (Carda‐Auten et al., 2022). The aims of the current study are to examine the carceral system’s ability to deliver healthcare services and a safe residence for people incarcerated during the outbreak of the COVID-19 pandemic and to better understand the perspectives of people directly affected by the system.

## Methods

Broadly, we recruited participants for our study from non-profit reentry groups that service persons returning home from incarceration. We enrolled the first 12 volunteers who were (1) adults and (2) incarcerated during the target period of March 2020, and conducted in-depth interviews about their living environments and interpersonal relationships. We focused our questions on health and healthcare but used open-ended questions that allowed participants to relay what they thought was important.

### Sample

Participants were formerly incarcerated individuals (*N* = *12*) who spent at least 10 days inside a NYC jail and were released on March 1 st, 2020 or after. Participants came from four different NYC jail facilities. Table 1 describes participant demographics, including the amount of time each participant spent inside a jail. Inclusion criteria ensured participants could discuss experiences of incarceration during the beginning of the COVID-19 pandemic.

**Table 1:** Demographic characteristics of participants

	**N**	**%**
Age	Mean = 38.33	sd = 9.8
Gender		
Men	11	92
Women	1	8
Race/Ethnicity		
African American	6	50
White	2	17
Puerto Rican	1	8
Not specified	2	17
Length of stay		
15 to 30 days	2	17
30 to 45 days	0	0
45 to 60 days	1	8
60 to 75 days	1	8
75 days or more	7	5
^a^Missing	1	8
Correctional Facility		
AMKC	2	17
MDC	5	42
RMSC	1	8
VCBC	1	8
Missing	3	25
Health Status^b^		
Mediocre or poor	1	8
Good	4	33
Very good	3	25
Excellent	3	25
Missing	1	8

^a^Participant verbally screened prior to interview to determine eligibility. However, confirmation of this was not recorded during the actual interview

^b^Participants self-reported their health status by selecting which category they felt best represented their current health

We enlisted the assistance of the reentry organizations,The Fortune Society and Exodus Transitional Community, based on prior experiences conducting jail-based research and health promotion. These organizations provide essential support to formerly incarcerated persons and acted as our intermediaries. In one instance, an interviewer called upon the intermediary to connect a participant to mental healthcare resources based on their interview responses. The study was approved by the Institutional Review Board of The City University of New York. 

We made three accommodative changes during our recruitment process. We increased the participation incentive from $25 to $50 (and paid out the difference to earlier participants), clarified on the recruitment flyer that participants could phone in to the Zoom call, and offered to communicate via text rather than email. Reentry staff assisted in disseminating recruitment flyers advertising the study, eligibility criteria, and researchers’ contact information. Staff at Fortune Society also invited the researchers to attend weekly meetings where the researchers purposively recruited an initial sample of 11 participants based on study criteria. One additional participant was recruited from Exodus via a recruitment flyer disseminated by a staff member. Their responses confirmed that no new information was being generated prompting the end of recruitment. An additional 15 potential participants expressed interest in the study. Three did not meet eligibility criteria and 12 were lost to follow-up.

All participants who completed an interview were initially sent a $25 Amazon or Target gift card to their email. Participants recruited later were sent a $50 gift card either by text or email. Interviews were conducted between August and December 2020 and recorded via Zoom in all cases but one where a phone call was preferred by the participant. Audio recordings were professionally transcribed and subsequently reviewed by the first and second authors for quality assurance.

### Data collection and analysis

The interviews allowed in-depth exploration of participants’ experiences with programs and services in jails, including interactions with the medical system: accessing medical care, patient-provider interaction, and resulting treatment. Participants were also asked about the new Department of Corrections (DOC) safety precautions for COVID-19. Labor conditions and relationships with others emerged as prominent themes and were therefore included in our analysis. Saturation was reached across interviews. The interview guide (Appendix A) was reviewed by researchers with extensive experience conducting research in New York City jails.

Data analysis followed an inductive, grounded theory approach and was iterative (Ritchie et al., 2014) and Dedoose (Version 9.0.107), a qualitative data analysis software, was used to code transcripts. Code definitions were refined throughout the periods of data collection and data analysis. The first three authors reviewed the transcripts while exploring emerging themes. Our codebook was then reviewed by the last author and adjusted based on their feedback. Each author first applied the codebook to the same two transcripts to ensure consistency. During the coding process of the remaining interviews, frequent check-ins between interviewers and coders, independent coding of each interview by at least two authors, and regular meetings during coding helped mitigate the potential for individual biases. Based on this coding process, we identified six prominent themes from 24 codes and detail three of them below. We chose to focus on these three themes as they are at once underrepresented in the current literature and uniquely suited to the strength of these data, drawing on the internal world of the incarcerated individual.

## Results

### Navigating relationships

***…**** with corrections officers.* While nuances emerged, overwhelmingly participants described their relationship with correctional officers (COs) in terms of an absence of humanity, a theme that persisted before the onset of COVID-19 and worsened afterwards. As one participant expressed, “*There was a lack of compassion. It was more or less, you know, you're an inmate just deal with it*.” (P8).

Our formerly incarcerated participants suggested that staff, and wider society, saw disrespect as just punishment. Some perceived conflict between that orientation and the system’s purported goal of rehabilitation. Others reported how the contentious relationship with COs and resulting treatment could actively worsen mental and physical well-being in the face of persistent lockdowns and other COVID-19 related stressors. In one example our participant describes COs interrupting sleep:

*“…the level of disrespect that some of these COs have, it’s immeasurable. A lot of the times, they abuse their power. They would walk into the house, jingling their keys around, waking people up in the middle of the night, making sure they slam the door on the way out. Just the type of behavior that can trigger an inmate to really want to snap. It’s bad enough that we're in there, the least that you can do is let us sleep.... it was really, really hard to sleep comfortably. It would give you PTSD. If you didn't have PTSD going into jail, you'd have PTSD coming out because they would interrupt your sleep.” (P8)*

Still, participants recognized nuances in the role played by COs. For example, they relied on COs for protection during violent altercations between residents. What results is a complicated but especially consequential relationship with the potential for either antagonism or favoritism. Officers developed a rapport with some residents while others felt on the defensive, trying to protect themselves from co-residents as well as officers.

Participants described their experiences of spring 2020, trying to piece together the unfolding information about COVID-19 within a context that habitually restricts the flow of information, creating a culture of secrecy. According to several participants, the need for information took a toll on individual psyches and on the collective living environment. As one participant describes:

“*It was very scary, like you wouldn’t know what was going on and people were freaking out. It… caused a lot of chaos. You didn’t know if you’re going to die or not... Just that worrying alone would just kill you.” (P5)*

In addition to the culture of secrecy, incarcerated people saw COs as a potential vector of the COVID-19 virus. Officers came in and out of facilities every day with many residents noting a lack safety of precautions. Instead, they saw apathy on the part of COs with some coming into work while feeling sick and expressing disregard for spreading disease. Participants also questioned the CO’s financial incentives. In conversations with residents, officers described receiving overtime pay when they were sick and because of this continued working without masks in hopes of being infected again.

***…****with co-residents.* Among the incarcerated persons, participants described a heightened intensity to their interpersonal relationships. This could mean increased violence due to the stress of the pandemic. For example, one participant discussed the frequency of alarms during this time and the toll it took on residents mental health. However, it could also mean a heightened sense of solidarity. As one participant concisely stated, *“If one falls, the rest of us fall.”* (P2) This sentiment was salient among participants. Disputes became less important when everyone involved thought their lives were on the line. Residents stopped much of the infighting and focused on maintaining their collective safety.

Residents also used this solidarity to disrupt the culture of secrecy. They relied on one another to share information and to help access resources like medical care. Participants described “banging on doors” and “rioting” to ensure co-residents received the medical treatment they needed. In one of the more harrowing experiences shared in our data, this participant relayed a dramatic, even violent form of solidarity, enacted to gain healthcare access:

*“He was doing everything he could to get the medical attention that he needed. And we was with him. And it went as far as where we had to punch him in the face to make him bleed.... We had to, to make him bleed, to make the COs and the captain and the medical [recognize that], ‘Okay, yeah. He needs medical treatment.’” (P3)*

To some extent, the sense of togetherness was felt among both residents and staff. While the most frequent bonds of solidarity that were reported were among incarcerated persons, solidarity could extend to correctional staff at times. One participant reflected on this compassion for COs:

*“[A new CO] just came from Brooklyn House and he was telling them [other COs] about COVID-19, he was scared. And they didn't tell him nothing. He started crying. So, we didn't even get mad at him.... Then they took the CO out from the house. For what? Well, we found out…he's not supposed to be bold in crying. That's showing he’s weak. We didn't care. We gave him mad respect because he was honest. He was scared. We felt for him.” (P3)*

### Seeing a doctor

People who are incarcerated disproportionately bear the burden of chronic disease, making healthcare inside jails an important and necessary component of the experience of incarceration (Brinkley-Rubinstein, 2013; Freudenberg, 2001). Our participants’ experiences with medical staff varied. Many of our participants reported substantial barriers to care during the COVID-19 outbreak, including inadequate medical staffing and relationships with medical personnel that embodied a lack of compassion and respect, indifference to their stated medical needs, and inadequate treatment. These experiences led some to only seek treatment for urgent medical issues. One participant remarked that they refer to the medical professionals as veterinarians because their treatment was akin to that of animals:

*“We used to have this joke when I was in prison. We’re going to see the veterinarian. These people who work on animals not people. And that was like a known thing around prison, everybody saying, ‘We’re going to see the veterinarian.’ Because like you went in there with something and you came out more worse than you went in there.” (P4)*

However, others with severe or chronic medical conditions were better able to build good relationships with their healthcare providers and were grateful for the quality of care they received. Participants expressed that building these relationships was likely possible because their chronic conditions required regular care and therefore consistent interaction with medical staff. This favoritism could result in privileged access to healthcare services and resources that were particularly useful during the height of the pandemic, but could lead to questions from fellow residents. For example, as one participant describes:

*“And if I wasn’t so close with some of the nurses there I wouldn’t have been able to get a mask. And then it was hard to wear the mask because then again everybody was like, ‘How the hell did you get a mask?’...I’m still alive because of that relationship– the rapport I kept with them [healthcare providers].” (P5)*

Unsurprisingly, the medical system had issues adapting to the COVID-19 pandemic. Participants reported an inability to get tested for the virus. Some were told they were going to be tested, but only underwent a temperature check, even when testing was specifically requested. One participant expressed discontentment when he was placed in a crowded room with close to 40 other men who also only received temperature screening. Even those who were quarantined as a result of showing symptoms consistent with COVID-19 reported an inability to get tested or to speak with a provider. One participant who displayed symptoms discussed his experience, saying:

*“They put us in a room, and then they had us quarantined, but we didn’t go to no medical [ward], no nothing. Like nobody came to see us at all, and I found that really strange because I’m like, yo, how is it that we’re quarantined, but yet we ain’t seen no doctor… that’s medical negligence. That’s not professional.” (P6)*

### Labor in jail

Many of our participants were eager to discuss labor conditions in the carceral system. They describe a system that was disorganized and coercive, unable to adapt in the face of the virus, and ended up a significant source of exposure risk. The use of these coercive practices magnified the danger to residents, particularly during an infectious disease outbreak.

Jails and prisons in the United States are exempt from the Fair Labor Standards Act, leaving incarcerated persons vulnerable to underpayment and coercive employment practices. Jail staff might coerce labor by threatening to remove good-behavior time, which could accumulate to secure early release. One participant discussed his experience working in the jail and his interactions with COs:

*“We didn't get paid, so I refuse to go and continue going to work. And eventually, they [COs] tried to tell me that they were going to take my good time from me if I didn't go, even though I wasn't being paid, and I told them no. I like studying the law and I had a lot of free time, so I told them they couldn't do that, but a lot of other people they went, they weren't paid, because they were afraid, they were gonna lose their good time. I said no, you don't pay me, I don't go.” (P11)*

Furthermore, newly implemented safety precautions meant more frequent cleaning and despite the intent of these safety measures, the COVID-19 outbreak meant this cleaning was high-risk – often without PPE. As one participant described:

*“...they came over and asked, ‘We’re asking for volunteers for cleaning the cell that whomever had corona in it.’ And then they were saying, ‘Yeah, we’re going to pay you like $100 for the day if you go over there and clean it. And we’d give you the proper cleaning equipment.’ So, when we went over there, they didn’t even give us gloves. They just basically gave us a bottle of bleach spray and the scouring powder and we were supposed to touch stuff in there with no proper bodily protection, no suits or nothing to clean this thing… They were asking you to risk your life for nothing.” (P5)*

In sum, participants described being coerced into unsafe working conditions without any compensation for their labor. Moreover, they lacked the necessary information regarding virus transmission or sequelae to make an informed decision about whether to engage in this type of work or the associated risks. Additionally, participants knew that if they were to become sick it would be difficult to access medical care, at best; at worst, the disease could lead to death.

## Discussion

This study elicited the views of twelve individuals recently released from jail who were incarcerated early in the COVID-19 pandemic for their perspectives on labor, safety, medical care, and interpersonal relationships in the NYC jail system amidst the unfolding pandemic. Their experiences relay the difficulty that the carceral system had with acting to protect health and safety within structures built on power dependence and charged to disempower. In his discussion of social relations, Robert Emerson describes power as dependency, when one depends on another for some material or psychic need (Emerson, 1962). This power increases with the superordinate’s endorsement (the external legitimacy of their power) and decreases when the subordinate has alternative means to access the need (Emerson, 1962; Ford & Johnson, 1998). Carceral institutions are an extreme example of power dependency. In correctional systems, staff, including guards and medical personnel have unfettered endorsement from jail administration, government officials, and even the public who are socialized to accept carceral norms. Similarly, incarcerated persons have limited alternatives to adhering to staff demands. They are inherently the subordinate party, a notion that arises repeatedly throughout this study and contributes to their relationships with staff and with each other. These data complement epidemiologic data, showing increased rates of transmission, and recent findings on the resource constraints reported by jail staff, to help guide jail health policy in three particular areas: sanitation, health communication, and healthcare delivery (Carda‐Auten et al., 2022; Jiménez et al., 2020). In addition, these three areas of interest all contribute to the inhumane care that most of our participants experienced. Addressing related health policies can not only improve the health and wellbeing of jail residents but also the treatment they receive from jail staff, including medical personnel.

### Key findings

#### Sanitation

First, sanitation is a heightened concern in a congregate residential setting, and even more so during an infectious disease outbreak. It is clear this carceral system’s infrastructure was not designed to protect residents. Overcrowding provided ample opportunity for infectious disease spread and the residential spaces lacked professional cleaning. Much of the cleaning that did occur was through the participants’ coerced labor and without proper protective equipment and cleaning supplies.

The system responsible for these environmental conditions is rooted in the inherent power-dependence relations inside jails and the dynamic of disempowering persons held under state control. As suggested in recent work on jail health, policies could stipulate that incarceration spaces meet public standards dictated, monitored, and enforced by epidemiologists and other public health experts in disease transmission and occupational and congregate safety, akin to external standards set for healthcare in schools (Carda‐Auten et al., 2022). Instead, our participants described a system wherein sanitation and access to PPE and healthcare were subsumed into the purview of a correctional system charged with revoking their autonomy and often regained only through interpersonal relationships with individual guards and medical personnel. Cleaning was coerced and payment withheld by a dense bureaucracy. Masks were reserved for those with whom staff developed a rapport. Healthcare was delayed until one could command the concern of an individual institutional actor. These are basic principles of disease mitigation but were left up to the jail system and then subsumed within the hierarchies of interpersonal power.

#### Communication

Second, residents lacked independent access to up-to-date and trusted public health information. This further cemented the guards’ power as residents depended on them for this valuable information. Yet participants reported being denied this information, being told for example not to worry about people wearing biohazard suits outside of their windows. The lack of information caused chaos and emotional distress. Residents struggled to piece together any information they came across, through windows, from officers who chose to share or chose to allow television news, or from word-of-mouth through one’s own or a coresident’s non-incarcerated friends and family. The distrust in staff and guards fostered a needed sense of solidarity among residents – they trusted one another for information and relied on each other for support during an unprecedented time.

Again, these standards fail to live up to the principles built up over centuries about communication regarding infectious disease. Trusted information is crucial to developing buy-in with the measures needed to prevent disease transmission, like mask-wearing, handwashing, and vaccine acceptance, all of which become more important in a setting where co-residents’ health are heavily intertwined. More trustworthy information could be disseminated through a “bottom-up” approach, in which communication is developed and delivered in partnership with the affected community, for example by cultivating the inherent bond between formerly incarcerated individuals by hiring them as public health communicators and care givers (Porat et al., 2020). These individuals are outside the confines of the correctional system, who are not tasked with controlling everyday aspects of residents’ lives and therefore do not benefit from the use of similar coercive tactics employed by carceral staff.

Information that inspires action fosters within the listener feelings of autonomy (I have the power to affect positive change), competence (I have the know-how and tools to affect positive change), and relatedness (I feel understood, respected, and cared for) (Van den Broeck, et al., 2010; Deci & Ryan, 2000; Vansteenkiste et al, 2008). Incarceration counters these principles in many ways: it is designed to actively usurp individual autonomy, condemns the individual’s decision-making capacity, and isolates them from family and friends, instead surrounding them with strangers and guards. Carceral systems may not be capable of developing or delivering public health messaging to its residents on their own.

#### Health care

Finally, better health care is critical to mitigate and monitor disease spread, manage symptoms, and protect the health and wellbeing of people whom the state incarcerates. Several interviewees reported that officers withheld needed approval to access a medical clinic, yet another instance of the carceral system’s monopoly of power over jail residents and a consequence of the relationship between residents and jail staff. These power dynamics also prevented several residents from getting the care they needed as many reported being disbelieved and dehumanized by medical professionals. Improved policy would allow consistent access to accurate biological tests (rather than symptom screenings) for communicable disease, on demand and at regular intervals; easily accessible vaccines as needed, and the capacity to verify one’s vaccine history. Better policy could also ensure that medical care meets basic professional standards of clinical treatment guidelines. Yet the structure of incarceration which fosters antagonistic relationships and supports a hierarchy of power is not well-equipped to ensure this from within.

#### Inhumane care

The unsanitary environment, lack of communication, and poor health care have all been cited as common issues across U.S. jails. These conditions, rooted in the power imbalance between staff and residents, contribute to the oppressive nature of incarceration. Individually, they foster feelings of dehumanization. Together, they create an environment that becomes especially dangerous during an emergency health crisis.

The lack of sanitary facilities alone constitutes inhumane conditions. This inhumanity is compounded by the coercion of residents to clean them without appropriate protective gear, directly compromising their physical safety. This deliberate endangerment was magnified by an acute lack of communication that left residents uninformed and unable to protect themselves from the outbreak. Ultimately, this system which revokes both physical and bodily autonomy led to many residents becoming ill. Even then, testing and medical treatment were difficult to access. These issues coupled with officers’ indifference to infection exposure further compromised the health of residents, solidifying the subordinate nature of incarceration. Addressing these three critical areas through policy is essential for counteracting the destructive nature of power-dependent relationships and affording residents humane care inside carceral facilities.

#### Policy recommendations

In each of these three arenas, sanitation, communication, and medical care, the jail system is tasked with carrying out these policies, but implementation is subsumed into the overarching process of disempowerment, which can be counterproductive for the policy goal. The data collected through our interviews illustrate a paradox at the heart of carceral healthcare that complicates the potential for the institution to protect their health in an emerging pandemic. The system is designed to disempower – taking away autonomy over employment, decisions to protect oneself, hygiene, nearly every aspect of daily life. All of these dimensions become the domain of guards who are hired, trained, and socialized to use their power over incarcerated people’s basic needs as tokens of punishment and coercion and with whom residents harbor deep-rooted distrust. Even medical care, ostensibly there to protect one’s wellbeing, is subsumed both in access and in content to this power-dependent hierarchical relationship. This led to operational patterns that undermined effective messaging, self-protection, and adequate access to care.

Such a system largely failed to adapt to the challenge of protecting incarcerated persons during the COVID-19 pandemic. Jails and prisons nationwide continued to proliferate with the disease, with nearly 650,000 cases and almost 3,000 deaths reported in 2022 (Brinkley-Rubinstein & Nowotny, 2022). In our data, respondents described how masks, soap, testing, and healthcare were allocated within a structure marked by a sense of scarcity and favoritism rather than in accordance with the emerging public health principles of COVID-19 prevention. The relationships inside, positive and negative, shaped their experiences with COVID-19 and at times, dictated their health outcomes. As a result, incarcerated people suffered psychological and physical trauma, and many died.

There are two kinds of policy progress that could meaningfully address this paradox. One would be to bring in experts that are external to the process of incarceration, who are not trained and employed within a paradigm of disempowerment, to oversee the health and wellbeing of incarcerated persons. Building on the bonds of solidarity described by our participants, employing formerly incarcerated individuals trained in these areas could help further the acceptance and trust needed to protect incarcerated persons during an infectious disease outbreak. Experts could include public health messengers for health communication; cleaning crews and human resource consultants for sanitation and employment; and all levels of healthcare providers for outreach within, removing the capacity for officers to overrule their needs. These external entities are held to academic and health standards rather than the professional standards of law and order to which guards adhere. This would allow a greater sense of power and autonomy among residents and thereby limit the effects of this relational power imbalance within the realm of public health. Another is to reduce the number of people incarcerated. The United States incarcerates a far higher percentage of its population than any other country in the world, including many who were never convicted of a crime and were never accused of violence. We incarcerate people for failure to pay child support, or for allegedly stealing a backpack (Gonnerman, 2014). We then subject those persons to a residential setting with easily transmissible disease and little meaningful recourse for instances of interpersonal inhumanity on behalf of those authorized to mitigate disease. Reducing the number of people incarcerated by this system might be necessary, but it is not sufficient to redress structural failures. As such, these two forms of policy progress are not mutually exclusive.

## Limitations

Our data were limited, in part, based on our recruitment primarily through reentry groups. These groups play an important role in previously incarcerated persons’ reintegration with their communities and their ability to access necessary services. As a result, our participants’ experiences may be more positive than the general population, due to their connection to post-incarceration services. We believe that this positioning results in relatively conservative conclusions. Additionally, only one person who identified as a woman was recruited, as most reentry group members and most jail residents, at the time were men. Lastly, this study focused solely on the experiences of those incarcerated in NYC specifically and we cannot speak to its generalizability.

## Conclusions

Across a variety of domains, the power differential inherent to incarceration and the antagonistic relationships formed as a result impeded the jail system’s capacity to protect and promote the health and wellbeing of incarcerated persons as this novel pandemic emerged. Residents lacked the trust necessary for effective public health communication and staff lacked the trust needed for effective monitoring and intervention. Disorganization and dehumanization hindered the ability of medical staff to deliver healthcare. The public health consequences of these failures are heightened by disproportionately high rates of incarceration among Americans as a whole and even more so among the socio-economic and minority identity groups of Americans who shoulder the greatest burden of both disease and incarceration, including the communities to which our participants ultimately returned. Our study data underscore the consensus conclusions emerging from the fields of public health and public policy about the inherent challenges of incarceration, and correspondingly, the benefits of decarceration (National Academies of Sciences & Medicine, 2020).

## Data Availability

Qualitative data that support the findings of this study have been provided in the manuscript and are stored on a locked hard drive owned by the primary author.

## Appendix

### Interview guide

Prior to the start of the interview, participants were briefed on the scope of the interview and the researchers’ interest in understanding participants perspectives in jail while they resided there during the COVID-19 pandemic.

1. To start, can you tell me a little bit about yourself?
2. What issues come to mind when considering the jail system’s ability to respond to the coronavirus for people who are incarcerated?
  a Can you tell me about the accommodations for sleeping, eating, recreation or exercise, and hygiene like showers?
3. Can you describe an experience when you needed medical care while you were incarcerated?
  a Probe about providers, wait times, supplies, environment, medical components.
  b Probe about perceptions of the provider- did they appear to care; did they listen and provide necessary information for follow up care?
4. How seriously would jail staff take your concerns if you thought you needed medical care?
  a Can you describe any hesitations that come up when asking for care?
5. The Dept of Corrections is taking some steps to address the virus. For example:
  - Displayed posters and videos with safety tips
  - Distributing masks and hand sanitizer
  - No visits from family and friends for a while
    a  Do you think the facilities will be able to do this well, and to stop the spread of the virus?
6. If you could decide what to do, for example if you were the governor, what do you think would be the most important steps to ensure that incarcerated people get high quality healthcare when they need it and can protect themselves from disease?
7. Could you tell me about returning to your community during this pandemic?
  a Have you needed healthcare? Did you get it? Have you been tested for the virus? Do you have a main focus or issue at the moment?
8. What have I not asked that might be important to ask others?
